# O6-methylguanine-DNA Methyltransferase Promoter Methylation in Patients with Rectal Adenocarcinoma After Chemoradiotherapy Treatment: Clinical Implications

**DOI:** 10.4274/balkanmedj.galenos.2019.2018.12.93

**Published:** 2019-08-22

**Authors:** Jaime A. Oliver, Jaime Gómez-Millán, Jose A. Medina, Laura Cabeza, Gloria Perazzoli, Cristina Jimenez-Luna, Kevin Doello, Raúl Ortiz

**Affiliations:** 1 Center for Cancer Research and Cell Biology, Queen’s University Belfast, Belfast, UK; 2 Institute of Biopathology and Regenerative Medicine, Center of Biomedical Research, University of Granada, Granada, Spain; 3 Department of Radiation Oncology, Universitary Hospital Virgen de la Victoria, Málaga, Spain; 4 Department of Anatomy and Embryology, University of Granada, Granada, Spain; 5 Biosanitary Institute of Granada (ibs. GRANADA), SAS-Universidad de Granada, Granada, Spain; 6 Medical Oncology Service, Universitary Hospital Virgen de las Nieves, Granada, Spain

**Keywords:** Chemoradiotherapy, O6-methylguanine-DNA methyltransferase, rectal adenocarcinoma

## Abstract

**Aims:**

To analyze the clinical relevance of O6-methylguanine-DNA methyltransferase in rectal adenocarcinoma treated with chemoradiotherapy followed by surgery.

**Methods:**

Tissue samples from 29 rectal adenocarcinoma patients were obtained after chemoradiotherapy. O6-methylguanine-DNA methyltransferase promoter methylation status was established by methylation-specific polymerase chain reaction. O6-methylguanine-DNA methyltransferase protein levels were determined by immunohistochemistry. Clinicopathologic variables, including treatment regression grade, recurrence, lymph node invasion, and stage and differentiation grade of the tumor, were determined.

**Results:**

The O6-methylguanine-DNA methyltransferase gene promoter was methylated in 81.5% of samples. Most patients (88.9%) showed low O6-methylguanine-DNA methyltransferase protein expression. O6-methylguanine-DNA methyltransferase methylation status was not correlated with any of the clinicopathological variables determined in rectal adenocarcinomas selected for chemoradiotherapy.

**Conclusion:**

O6-methylguanine-DNA methyltransferase methylation status is not correlated with clinicopathologic variables examined in rectal adenocarcinoma selected for chemoradiotherapy, although its role as a biomarker awaits further investigation.

Patients with rectal adenocarcinoma stage II-III are usually treated with preoperative chemoradiotherapy based on 5-fluorouracil or capecitabine. However, little data on molecular biomarkers for the prognosis and treatment response in colorectal cancer has been obtained (1). The enzyme O6-methylguanine-DNA methyltransferase (MGMT), which eliminates methyl groups in the O6-guanine position avoiding G:C to A:T transitions, has also been related to colorectal cancer (2,3). MGMT prevents cell death due to cytotoxic drugs by repairing DNA, but it can be silenced by epigenetic methylation (4). Loss of MGMT expression has been detected in colorectal cancer and associated with G to A transition in the p53, K-ras, and PIK3CA genes (5). Previous studies suggested that MGMT promoter methylation status was related to glioblastoma treatment failure (6). In this study, MGMT expression and MGMT promoter methylation status were evaluated in rectal adenocarcinoma patients after chemoradiotherapy treatment in order to determine their status and relevance as prognostic biomarkers.

## MATERIALS AND METHODS

### Clinical history and tissue samples

Twenty-nine rectal adenocarcinoma patients (stage II-III) who were candidates for preoperative chemoradiotherapy were recruited after they gave informed consent (Biomedical Investigation Ethic Committee; Servicio Andaluz de Salud). All patients were evaluated before treatment (physical examination with a digital rectal examination, colonoscopy and biopsy, chest X-ray, abdominopelvic scan and/or endorectal ultrasound, and magnetic resonance image of the pelvis). These patients were treated with pelvic radiotherapy (46-50 Gy in 2 Gy fractions) and intravenous 5-fluorouracil (5-day cycles of 500 mg/m^2^ 5-fluorouracil every 21 days) or capecitabine (4 cycles of 1250 mg/m^2^ capecitabine every 12 h for 14 days) followed by surgery (total mesorectal excision) 6 weeks after chemoradiotherapy. Tumor samples were obtained from each patient from endoscopic biopsy before chemoradiotherapy. The chemoradiotherapy response was staged histopathologically on the basis of tumor regression grade (Mandard’s classification: grade I and II = complete/partial regression and grade III, IV, or V = no regression) (7). Two expert pathologists evaluated an intra-operative sample after chemoradiotherapy. Demographic data (sex and age) were obtained. In addition, clinicopathological variables, including tumor differentiation grade, tumor stage, treatment regression grade, recurrence, and lymph node invasion, were analyzed.

### Methylation-specific polymerase chain reaction and immunohistochemistry

DNA was extracted from paraffin-embedded tissues by using a Chemagic MSM I robot (Chemagen, Germany, Baesweiler). Methylation patterns in CpG islands of the MGMT promoter were determined by methylation-specific polymerase chain reaction as previously described (8). Samples were classified as methylated (amplification product with M or both M and UM primers) and unmethylated (amplification with UM primers only). Immunohistochemical analysis was performed with a Dako Autostainer EnVision™ FLEX System kit (Agilent Technologies) and the results evaluated by two experienced pathologists. MGMT (1:50, Santa Cruz Biotechnology, Inc., Heidelberg, Germany) mAb was used as the label and 3.3'-diaminobenzidine as the chromogen substrate. Counterstaining was performed with hematoxylin (blue). As previously described by Oliver et al. (2), MGMT staining were scored and grouped as low (<50%) and high expression (≥50%).

### Statistical analysis

SPSS version 15.0 (IBM, Chicago, IL) was used for data analyses. Associations between promoter gene methylation status and clinicopathologic variables were analyzed by Fisher’s exact test. Results were considered statistically significant if p<0.05.

## RESULTS

**Figure 1:**
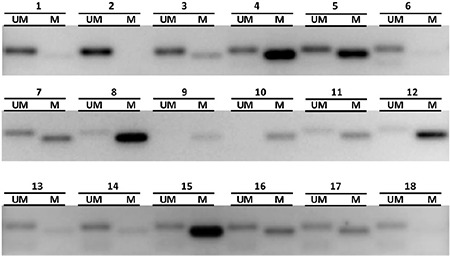
Methylation-specific polymerase chain reaction analysis of the O6-methylguanine-DNA methyltransferase promoter in rectal adenocarcinoma tissue samples. DNA was extracted by using a Chemagic MSM I robot (Chemagen, Germany, Baesweiler), denatured, and purified with an EpiTect Bisulfite kit (Qiagen, USA, Maryland). Primer sequences for the unmethylated reaction were 5`-TTTGTGTTTTGATGTTTGTAGGTTTTTGT-3` (forward primer) and 5`-AACTCCACACTCTTCCAAAAACAAAACA-3` (reverse primer) and for the methylated reaction were 5`-TTTCGACGTTCTAGGTTTTCGC-3` (forward primer) and 5`-GCACTCTTCCGAAAACGAAACG-3` (reverse primer). Polymerase chain reaction-amplified products were electrophoresed on 3% agarose gels, visualized by staining with ethidium bromide, and examined under ultraviolet illumination. The representative image depicts O6-methylguanine-DNA methyltransferase promoter methylation analysis of 18 samples. Patients with methylated promoters showed amplification in both unmethylated and methylated lanes or the methylated lane alone. The lack of a band in the lane corresponding to methylation-specific primers for rectal cancer sample 2, 6 or 18 reflects the absence of O6-methylguanine-DNA methyltransferase promoter methylation. M: methylated; UM: unmethylated

**Figure 2:**
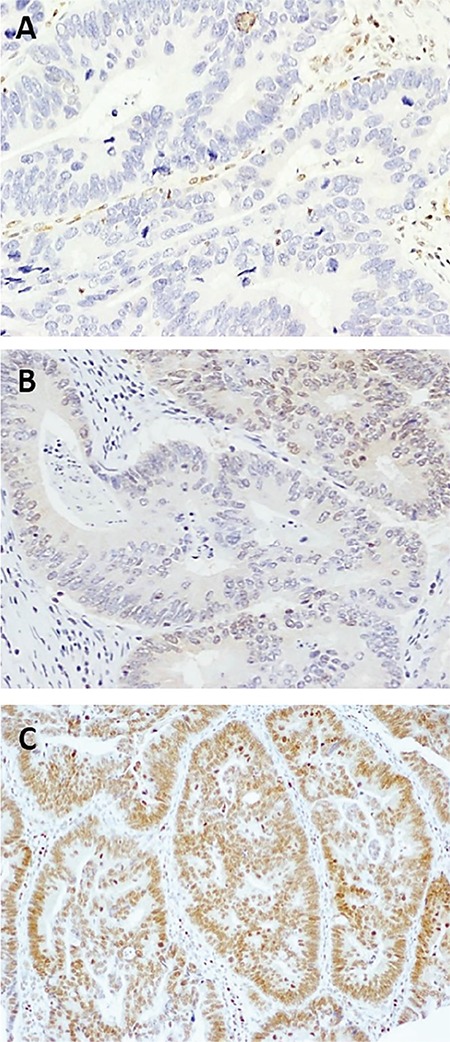
Immunohistochemical staining of rectal adenocarcinoma tissue samples with a mouse monoclonal antibody against human O6-methylguanine-DNA methyltransferase protein. Formalin-fixed, paraffin-embedded rectal cancer samples were stained with an antibody against O6-methylguanine-DNA methyltransferase (see Methods). O6-methylguanine-DNA methyltransferase staining of tumor cells was scored and grouped as low expression (<50%) (-, +, and ++ scores) and high expression (≥50%) (+++ and ++++ scores). The intensity of O6-methylguanine-DNA methyltransferase staining was scored as low or high. The figure shows representative photomicrographs of slides illustrating different percentages of O6-methylguanine-DNA methyltransferase expression. A tumor with no detectable O6-methylguanine-DNA methyltransferase expression (a); A positive tumor with low O6-methylguanine-DNA methyltransferase expression (˂50%) (b); A positive tumor with high O6-methylguanine-DNA methyltransferase expression (>50%) (20× magnification) (c).

The clinical patient characteristics are summarized in Table 1. The mean age was 64.43±12.24 years (range, 33-83 years); 75.9% (22/29) of patients were male and 24.1% (7/29) were female. The median follow-up period was 20.53±9.07 months. No patient died due to rectal cancer, and disease recurrence was observed in 13.8% (4/29). MGMT promoter methylation status could be determined in 93.1% of specimens (27/29). Of the 27 patients, the MGMT gene promoter was methylated 81.5% (22/27) (Figure 1). Immunohistochemical analysis (Figure 2) showed low MGMT protein expression in most patients (88.9%). Only 11.1% of patients showed high expression of MGMT. We also examined the association between MGMT promoter methylation and clinicopathologic features. MGMT promoter methylation status was not associated with sex, tumor differentiation, or tumor stage. Furthermore, no association between MGMT methylation and the clinicopathologic variables examined was detected (Table 2).

## DISCUSSION

The relationship between MGMT and colorectal cancer remains unclear, and results have been contradictory. Whereas Nilsson et al. (9) found a lower risk of recurrence in 5-fluorouracil-treated colorectal cancer patients with a methylated versus unmethylated MGMT promoter, Shima et al. (3) concluded that neither MGMT promoter methylation nor loss of MGMT expression is a useful prognostic biomarker. Sinha et al. (10) observed that MGMT methylation was associated with stage III in sporadic colorectal cancer cases. Recently, the methylation status of MGMT has been correlated with pathologic complete response in colorectal cancer patients (11).

Shalaby et al. (12) showed a good correlation between MGMT methylation and downregulation of its mRNA expression. Although these authors proposed MGMT methylation as a new biomarker to differentiate benign and malignant rectal tumors, no relation between MGMT methylation and clinicopathological features was detected. Sun et al. (13) showed that even the MGMT promoter methylation status of plasma-cell-free DNA was associated with a better tumor response. In addition, MGMT methylation levels in the blood were similar to those in rectal cancer tissues (12). Our results showed that the methylation status of the MGMT promoter was not associated with a better treatment response. However, the role of MGMT promoter methylation status as an early biomarker of colorectal cancer has not yet been established, despite several studies in colorectal adenoma and adenocarcinoma (14,15). In fact, Sideris et al. (16) showed recently that there was no association between the status of MGMT expression and pathological features, including response to neo-adjuvant therapy. Future research will be needed to elucidate the relationship between these biomarkers and rectal cancer treatment.

## Figures and Tables

**Table 1:**
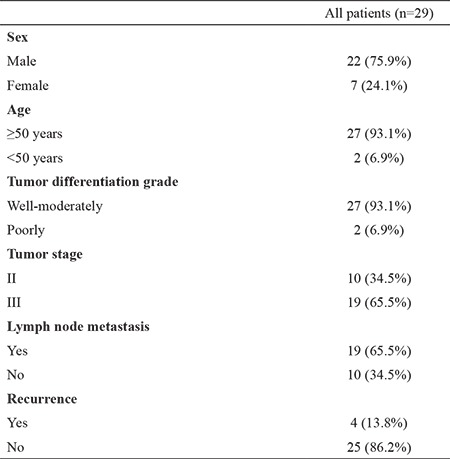
Characteristics of rectal cancer patients

**Table 2:**
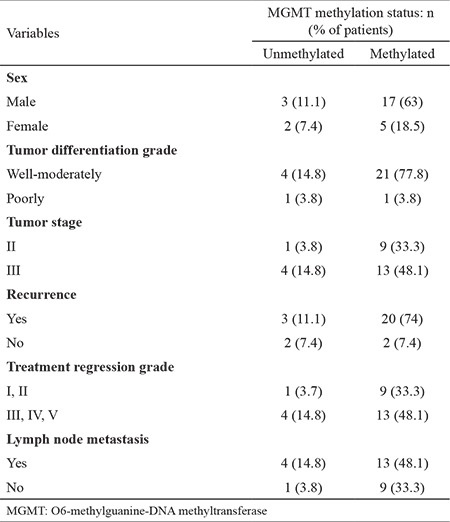
Correlation between MGMT methylation status and demographic and clinicopathologic variables
